# Novel Potentiometric 2,6-Dichlorophenolindo-phenolate (DCPIP) Membrane-Based Sensors: Assessment of Their Input in the Determination of Total Phenolics and Ascorbic Acid in Beverages

**DOI:** 10.3390/s19092058

**Published:** 2019-05-02

**Authors:** Nada H. A. Elbehery, Abd El-Galil E. Amr, Ayman H. Kamel, Elsayed A. Elsayed, Saad S. M. Hassan

**Affiliations:** 1 Chemistry Department, Faculty of Science, Ain Shams University, Cairo 11556, Egypt; nadaelbehery@hotmail.com; 2 Pharmaceutical Chemistry Department, Drug Exploration & Development Chair (DEDC), College of Pharmacy, King Saud University, Riyadh 11451, Saudi Arabia; 3 Applied Organic Chemistry Department, National Research Centre, Dokki, Giza 12622, Egypt; 4 Zoology Department, Bioproducts Research Chair, Faculty of Science, King Saud University, Riyadh 11451, Saudi Arabia; eaelsayed@ksu.edu.sa; 5 Chemistry of Natural and Microbial Products Department, National Research Centre, Dokki, Cairo 12622, Egypt

**Keywords:** 2,6-dichlorophenolidophenolate (DCPIP), total antioxidant capacity (TAC), ascorbic acid, potentiometry, ion-selective electrodes, flow analysis

## Abstract

In this work, we demonstrated proof-of-concept for the use of ion-selective electrodes (ISEs) as a promising tool for the assessment of total antioxidant capacity (TAC). Novel membrane sensors for 2,6-dichlorophenolindophenolate (DCPIP) ions were prepared and characterized. The sensors membranes were based on the use of either Cu^II^-neocuproin/2,6-dichlorophenolindo-phenolate ([Cu(Neocup)_2_][DCPIP]_2_) (sensor I), or methylene blue/2,6-dichlorophenolindophenolate (MB/DCPIP) (sensor II) ion association complexes in a plasticized PVC matrix. The sensors revealed significantly enhanced response towards DCPIP ions over the concentration range 5.13 × 10^−5^–1.0 × 10^−2^ and 5.15 × 10^−5^–1.0 × 10^−2^ M at pH 7 with detection limits of 6.3 and 9.2 µg/mL with near-Nernstian slope of −56.2 ± 1.7 and −51.6 ± 2 mV/decade for sensors I and II, respectively. The effects of plasticizers and various foreign common ions were also tested. The sensors showed enhanced selectivity towards DCPIP over many other phenolic and inorganic ions. Long life span, high potential stability, high reproducibility, and fast response were also observed. Method validation was also verified by measuring the detection limit, linearity range, accuracy, precision, repeatability and between-day-variability. The sensors were introduced for direct determination of TAC in fresh and canned juice samples collected from local markets. The obtained results agreed fairly well with the data obtained by the standard method.

## 1. Introduction

The organism’s status regarding cell damage is defined as oxidative stress caused by enhanced release of oxygenated free radicals [1]. These excessive amounts of free radicals arising physiologically during cellular aerobic metabolism, may lead to disruption of a living cell or to molecular and cellular DNA damage [2]. There are different types of oxygen-centered free radicals, collectively called reactive oxygen species (ROS). These radicals comprise superoxide anion radical (O_2_·^−^), hydroxyl (HO·), peroxyl (ROO·) and alkoxyl (RO·) radicals, and nitric oxide (NO·). Hydroxyl (HO·) and alkoxyl (RO·) free radicals are characterized by their high reactivity towards biomolecules, while, superoxide anion (O_2_·^−^) and nitric oxide (NO) have lower reactivity, as previously reported [3]. In addition to the aforementioned radical oxygenated species, singlet oxygen, hydrogen peroxide, or hypochlorous acid are also known as non-radical reactive oxygen species [4]. These oxygen- containing free radicals are implicated in cancer, cardiovascular diseases, cataracts, and dysfunctions of the immunity system which develop especially with age [5].

Oxidative processes can be inhibited by antioxidants [6,7]. Natural antioxidants, especially plant sourced ones, are now considered the best choice for safe food antioxidants. Epidemiological studies confirm that the rate of consumption of fruits and vegetables is inversely proportional to the development of oxidative stress-related disorders. This has been attributed to the presence of compounds with high antioxidant activity [8,9,10,11]. One of the main classes of bio-compounds found in natural sources are phenolics (flavonoids or non-flavonoids). These compounds are associated with the health benefits resulting from the inhibition of the oxidation of low-density lipoprotein [9,12,13,14,15,16].

Antioxidants have high chemical diversity and are found in complex mixtures in many real samples. It is difficult to successfully use any single separation technique for these complex mixtures. Moreover, the effect of a complex mixture of these antioxidants is almost inevitably different from the sum of the contributions of the individual antioxidants in the mixture [17]. According to these facts, the useful concept of total antioxidant capacity (TAC) has appeared. TAC considers the collective strengths of all antioxidants found in the sample instead of being concerned with the individual strengths of each antioxidant [18]. Different methods for TAC assessment are reported in the literature [6,19,20,21,22,23,24,25,26]. These methods vary widely in both the chemical concept and the numerical values obtained for TAC. Among of these methods, we may mention the ferric reducing antioxidant power (FRAP) [19], the Trolox equivalent antioxidant capacity assay (TEAC) [20], and the oxygen radical absorbance capacity (ORAC) [21]. All of the methods are carried out under widely different conditions and often produce quite different TAC assay results [22,23,24,25,26]. This makes any attempt to compare data obtained with different procedures ill-advised.

Recently, great attention has been directed to apply electrochemical tools to determine TAC. These tools have many numerous advantages. They are rapid, highly sensitive, and simple, requiring small sample volumes for analysis and low cost instrumentation. In voltammetry, a confirmed disadvantage results from the poor reliability of TAC values for the samples. This result unreliability is attributed to the strong dependence of the value obtained on the reaction mechanism at the electrode’s surface [27]. In amperometry, very strict control of the experimental conditions is required, so, the selectivity of the method depends on the specificity of the reaction between the oxidized or reduced form of the redox couple and the analyte [28,29,30].

Potentiometry as an electroanalytical tool offers advantages when compared with voltammetry or amperometry because it does not apply current or potential modulation [6,23,24,28,29,31,32,33,34,35]. The apparatus used in potentiometry is simple, easy to use, and the sample is viewed as the single independent variable [36,37]. Application of flow-through potentiometric measurements offers rapid and reproducible evaluation of TAC suitable for several aqueous plant extracts. TAC was estimated by a potentiometric tool via recording the potential generated from the reaction of K_3_[Fe(CN)_6_]/K_4_[Fe(CN)_6_] as a result of its interaction with the sample antioxidants upon using platinum electrode as a detector [38,39]. A potentiometric assay for non-invasively providing the skin oxidant/antioxidant balance, relied, as measured analytical signal, on the potential change of the ferricyanide/ferrocyanide [Fe(CN)_6_]^−3^/ [Fe(CN)_6_]^−4^ mediator system applied to the skin by means of a conductive gel. The change in the Pt electrode potential was caused by the shift in the oxidized form to the reduced form ratio of the mediator system. A decrease in the potential value indicates the antioxidant activity of the analyzed medium [40]. Another potentiometric antioxidant capacity assessment was reported in literature and applied in biological samples, food and drinks. It was based on the use of the free radical generator 2,2′-azobis(2-amidinopropane) dihydrochloride (AAPH) with K_3_[Fe(CN)_6_]/K_4_[Fe(CN)_6_] as a system mediator [41]. The sample underwent reaction with AAPH, so the antioxidants’ concentration is lowered, as a consequence of the interaction with peroxyl radicals generated from AAPH decay in phosphate buffer solution pH = 7.40 at 37 °C. Another potentiometric assay for TAC was reported using an I_2_/I^−^ mediator redox couple in 0.1 M, pH = 6.7 phosphate buffer. A Pt electrode was used as an indicator electrode [42].

2,6-Dichlorophenolindophenol (DCPIP) is a chemical used as a redox. DCPIP can also be used as an indicator for the assessment of ascorbic acid (vitamin C). If vitamin C, which is a good reducing agent, is present, the blue dye, which turns pink in acid conditions, is reduced to a colorless compound by ascorbic acid. Pharmacological experiments suggest that DCPIP may serve as a pro-oxidant chemotherapeutic targeting human cancer cells in an animal model of human melanoma; DCPIP-induced cancer cell death occurs by depletion of intracellular glutathione and upregulation of oxidative stress [43].

The goal of this work was to develop a method for the potentiometric determination of ascorbic acid and phenolic antioxidants, and to evaluate their contribution to the total antioxidant capacity of beverages. The method is based on the preparation and characterization of novel potentiometric ion sensors for measuring 2,6-dichlorophenolidophenolate ion (DCPIP). The membrane sensors consist of Cu^II^-neocuproin/2,6-dichlorophenolindophenolate ([Cu(Neocup)_2_][DCPIP]_2_) (sensor I), or methylene blue/2,6-dichlorophenolindophenolate (MB/DCPIP) (sensor II) ion association complexes in a plasticized PVC matrix. The proposed sensors revealed good performance features such as high sensitivity with reasonable selectivity for DCPIP ion, long term stability, and good precision. The constructed sensors were applied for accurate quantification of DCPIP dye under static and flow mode of operations. The method was applied for ascorbic acid and phenolic antioxidants determination, and their contribution to the total antioxidant capacity of beverages was evaluated.

## 2. Materials and Methods

### 2.1. Equipment

All EMF measurements were carried out at 25 ± 1 °C using pH/mV meter (Orion 720 SA-Cambridge, MA, USA). The measuring cell consists of DCPIP membrane sensors in conjunction with Ag/AgCl double-junction reference electrode (Orion 90-00-29). For flow-through measurements, a peristaltic pump (Ismatech MS-REGLO, Wertheim, Germany) and an injection valve provided with 4-port injection (Omnifit, Cambridge, UK) and a sample loop of 100 µL were employed. The potential readout signals were collected using data acquisition (eight-channel electrode-computer interface (Nico-2000 Ltd., London, UK) and Nico-2000 software).

### 2.2. Reagents and Chemicals

All chemicals and reagents were of analytical grade and used as received. Solutions were prepared with doubly distilled water. 2,6-Dichlorophenolindophenol sodium salt (DCPIP), high molecular weight poly(vinyl chloride) PVC, ascorbic acid, *o*-nitrophenyloctylether (*o*-NPOE), dioctylphthalate (DOP), tetrahydrofuran (THF) and neocuproin were purchased from Fluka (Ronkonoma, NY, USA). Sodium sulfate and methylene blue were purchased from BDH Chemicals Ltd. (Dubai, UAE). 2,2-Diphenyl-1-picrylhydrazyl (DPPH); L-ascorbic acid (Asc), catechol (Cat), caffeic acid (Caf), pyrogallol (Pyr), gallic acid (Gal) and ferulic acid (Fer) were purchased from Sigma-Aldrich (St. Louis, MO, USA).

For the preparation of (MB/DCPIP) and ([Cu(Neocup)_2_][DCPIP]_2_) ion association complexes, either methylene blue or [Cu(Neocup)_2_]^2+^ (10 mL, dissolved in ethanol, then added a few drops of 0.1 M CuSO_4_ solution) solutions were mixed with 1.0 × 10^−1^ M DCPIP solution (10 mL) and stirred for 15 min. Two colored precipitates were formed, respectively, filtered off, washed with doubly distilled water, and left to dry overnight at room temperature.

A 1.0 × 10^−1^ M stock DCPIP solution was prepared in doubly distilled water. Stock solutions of the antioxidants (0.01 M) were prepared by dissolving weighed amounts of the substances in an appropriate volume of water and they were kept in dark vials in a refrigerator. More dilute solutions were freshly prepared when needed by dilution of the stock solutions with water. Interfering ion solutions for selectivity measurements were prepared using 0.01 M solutions of the sodium salts of phosphate, citrate, chloride, bromide, thiocyanate, nitrate, nitrite, iodide, and acetate.

### 2.3. Membrane Preparation and Sensor Construction

The membrane-based sensors were prepared by dissolving the ion-association complex (either MB/DCPIP or [Cu(Neocup)_2_][DCPIP]_2_, 2 mg), the plasticizer (either DOP or *o*-NPOE, 133 mg), and PVC (66 mg) in THF (3 mL). This dissolved cocktail is poured into a glass ring (with an inner diameter of 22 mm) resting on a smooth glass sheet and then covered with a filter paper. The solution is left to dry overnight. The resulting membrane (0.5 mm thickness) was sectioned with a cork borer then glued with THF to a PVC tube. An Ag/AgCl wire (3 mm) was employed as an internal reference electrode and immersed in an internal filling solution consists of a mixture of equal volumes of 1.0 × 10^−4^ M 2,6-DCPIP and 0.01 M KCl. All membrane sensors were soaked in 1.0 × 10^−4^ M 2,6-DCPIP for conditioning towards calibration and stored at the same conditions when not used.

### 2.4. Direct Potentiometric Measurements

For static measurements, DCPIP membrane-based sensors in conjunction with a Ag/AgCl double junction reference electrode were immersed in a 50 mL beaker which contains 10 mL of 30 mM Tris buffer (pH = 7). Aliquots (0.5 mL) of standard 2,6-DCPIP solutions over concentrations ranging from 1.0 × 10^−4^–1.0 × 10^−1^ M were added and the potential of the following cell was recorded.

Ag|AgCl|sat. KCl|0.1 M CH_3_COOLi/sample solution||PVC membrane||1.0 × 10^−4^ M 2,6-DCPIP + 1.0 × 10^−2^ M NaCl solution|AgCl|Ag.

A calibration curve is constructed by plotting the change in the potential readings against log[DCPIP] anion. The obtained curve is used for determination of 2,6-DCPIP unknown concentrations under the same conditions.

### 2.5. Flow Injection Setup

**Figure 1:**
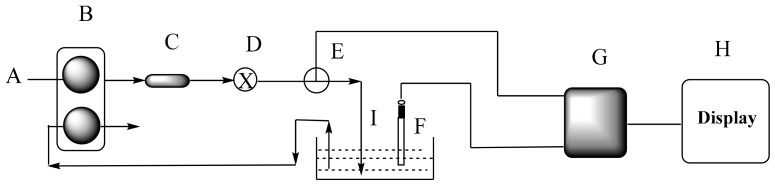
For the two channel FIA set up used for the determination of DCPIP: A, carrier tris buffer solution pH 7; B, peristaltic pump; C, pulse damper; D, sample injection valve; E, flow injection detector; F, reference electrode; G, data acquisition system; H, laptop computer; I, Petri dish.

For flow injection analysis a tubular detector was constructed by using membrane cocktail as previously mentioned [44]. The flow-injection manifold is shown in Figure 1.

The sensing membrane consists of 2 mg of different ion association complex, 66 mg of PVC and 130–133 mg of DOP plasticizer dissolved in 3 mL THF solvent. After that a window of 0.5 cm length and 2 mm width is made on a Tygon tube and a micro-dropper is used to drop from each cocktail on the surface of the window and left to allow slow evaporation of the solvent at room temperature forming a thin film with a thickness of approximately 0.1 mm. After that the tube is put in a pipette tip which is closed by a piece of Parafilm. The constructed sensors were conditioned in equal volumes of 1 × 10^−4^ M 2,6-DCPIP and 1 × 10^−2^ M KCl overnight. The sensing cell was 40 cm away from the injection valve, the end of the tube was placed in 100 mL beaker which contains the reference electrode.

### 2.6. Potentiometric Determination of Phenlic Antioxidants

A series of potentiometric monitoring was performed in which the sensor based on ([Cu(Neocup)_2_][DCPIP]_2_) (sensor I) was used as an indicator sensor. Test solutions containing single phenolic antioxidants (e.g., Cat, Caf, Pyr, Gal or Fer) or either binary mixtures (e.g., Cat + Fer, Fer + Pyr or Caf + Gal) or ternary mixtures (e.g., Cat + Fer + Pyr) were titrated with 1.0 × 10^−2^ M DCPIP solution. The concentration of each phenolic compound was determined (1 mol DCPIP = 1 mol Cat or Caf = 2 mol Fer; and = 2/3 mol Gal or Pyr).

### 2.7. Total Antioxidant Capacity Assay in Beverages

The usefulness of the constructed sensors is determined by their ability to measure the total antioxidant capacity (TAC) concentration in beverage samples collected from local markets. 2,6-DCPIP membrane sensor-based (MB/DCPIP) or ([Cu(Neocup)_2_][DCPIP]_2_) ion association complexes with the reference electrode were immersed in a 50 mL beaker containing 30 mL of the sample. The solution was titrated using two concentrations (1.0 × 10^−3^ and 1.0 × 10^−4^ M) of 2,6-DCPIP redox dye solution. After each volume addition, the potential readings were recorded. The equivalence point for the titration was calculated from the sharp inflection break or from first derivative curves, and the TAC concentration expressed was measured as mg/L ascorbic acid. 10–100 mg/L standards of ascorbic acid were taken as control and quantized by the same procedure described above. The results obtained from potentiometric titration were compared with the standard DPPH method expressed as mg/L ascorbic acid (AA) [45].

## 3. Results and Discussion

### 3.1. Performance Characteristics of the Sensors

**Figure 2:**
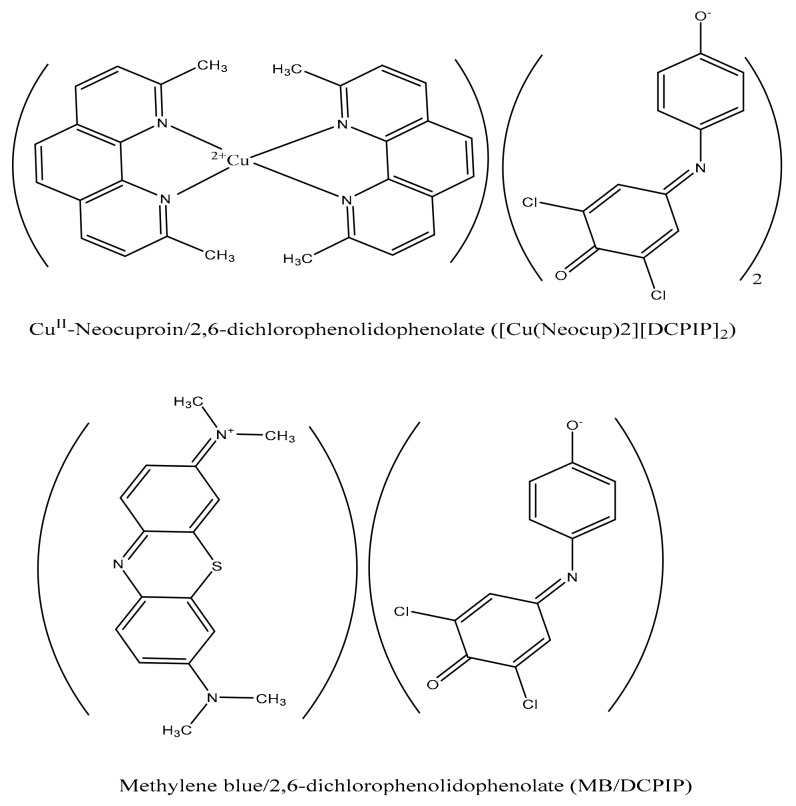
Structure of different ion pairs based on 2,6-DCPIP redox dye.

2,6-DCPIP anion reacts with [neocuproin-Cu^II^ and methylene blue dye] cations forming two 1:2 and 1:1 water insoluble ion association complexes, respectively (Figure 2). They were prepared and characterized as novel selective sites for 2,6-DCPIP in plasticized PVC matrix membrane sensors. The membrane composition was 66.2 wt.% plasticizer, 32.8 wt.% PVC and 1 wt.% the ion association complexes.

**Figure 3:**
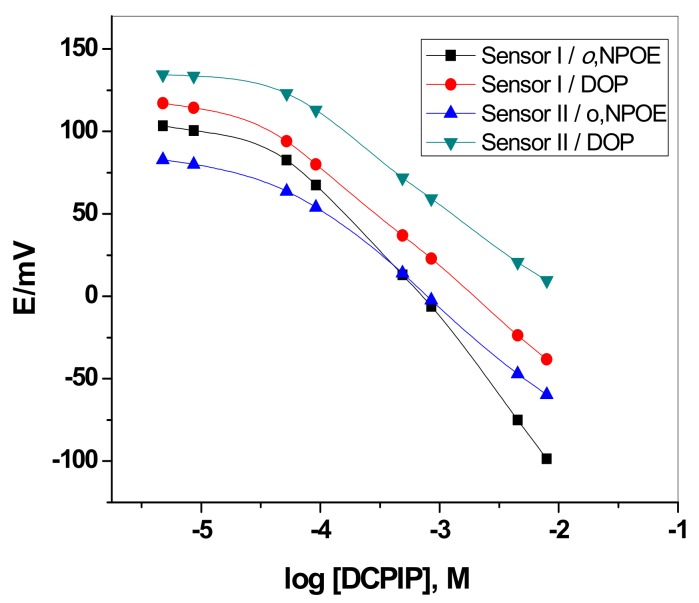
Effect of plasticizers on the potentiometric response of [neocuproin-DCPIP and methylene blue-DCPIP] membrane-based sensors.

For each carrier, membrane sensors (*n* = 4) were prepared and the performance characteristics were evaluated during 6 months according to IUPAC recommendations [46]. For optimizing the membrane composition, the effect of plasticizer was tested. The response characteristics of ion-selective sensors were influenced by the polarity of the plasticizer in the membrane. 2,6-DCPIP PVC matrix membrane sensors incorporating ([Cu(neocup)_2_][DCPIP]_2_) (sensor I) or (MB/DCPIP) (sensor II)with (DOP, ɛ = 8) and (*o*-NPOE, ɛ = 24) plasticizers were prepared and tested. For the ([Cu(neocup)_2_][DCPIP]_2_) membrane-based sensor, the slope and detection limit were decreased from −82.2 ± 1 to −56.2 ± 1.7 mV/decade and from 7.9 × 10^−5^ to 2.3 × 10^−5^ M upon using (DOP, ɛ = 8) instead of (*o-*NPOE, ɛ = 24). In addition, the calibration slope and lower limit of detection of (MB/DCPIP) membrane based sensor were −59.5 ± 1.4, −51.6 ± 2 mV/decade and 4.5 × 10^−5^, 3.4 × 10^−5^ M upon using *o*-NPOE and DOP plasticizers, respectively. It can be seen that membranes containing DOP gave more favorable lower detection limits and wide range of linearity than those containing *o-*NPOE plasticizer. The calibration curves of the previous sensors are shown in (Figure 3) and the performance potentiometric characteristics are listed in Table 1.

**Figure 4:**
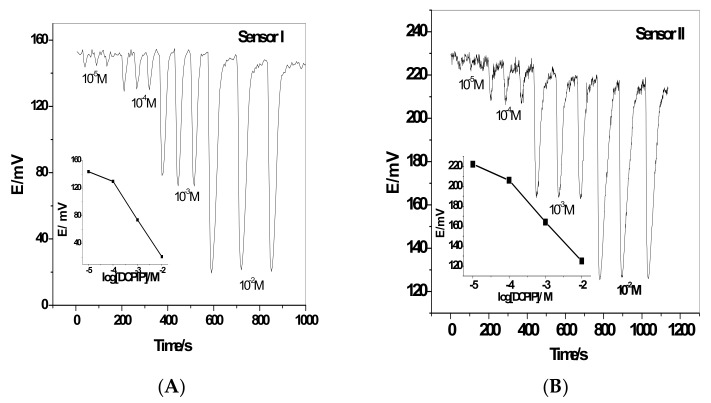
Signals obtained in triplicate for (**A**) sensor I and (**B**) sensor II. Conditions: carrier solution, 30mMTrisbuffer (pH 7.0), flow rate 3.5 mL/min; sample volume, 100 μL.

Measurement of 2,6-DCPIP under flow-through operation was carried out. Sub-Nernstian calibration slope over the concentration range of 9.8 × 10^−5^−1.0 × 10^−2^ and 1.0 × 10^−4^–1.0 × 10^−2^ M with a lower detection limit of 8.1 × 10^−5^ and 4.2 × 10^−5^ M and a slope of −42.36 mV/decade (*r^2^* = −0.974) and −33.3 mV/decade (*r^2^* = −0.984) (Figure 4). The obtained data under the optimized conditions of the flow-through measurements are shown in Table 2. The sampling rate was 42–51 runs per hour.

### 3.2. Method Robustness and Ruggedness

The ability of the present proposed method to remain unaffected by deliberate change of method parameters was also tested. Some of these are pH, sample size, carrier flow rate (in FIA) and injection volume were varied within a realistic range, and the quantitative influence of the variables is determined. In addition, four different sensor assemblies with two different instruments on different days were used for repetitive determination of different sample sizes of 2,6-DCPIP. Repeatability (within-day) and reproducibility (between-days) measurements showed potential variation in the range of 2–3 mV. These results revealed that the influence of these parameters was within the specified tolerance and the variations are considered within the method’s robustness range.

**Figure 5:**
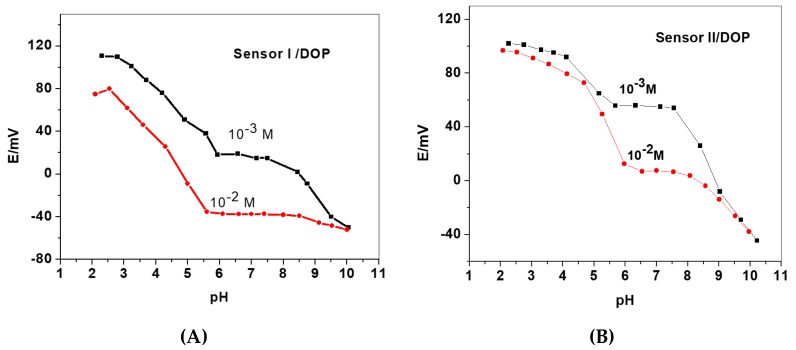
Effect of pH on the potentiometric response of (**A**) sensor I and (**B**) sensor II using DOP as a plasticizer

A study of pH effect for 2,6-DCPIP-based sensors were performed over the pH range 2–10 using two concentrations:1.0 × 10^−2^ and 1.0 × 10^−3^ M of this dye. The pH of these solutions was set by adding small volumes of concentrated NaOH and HCl solutions and that is by using combined glass-pH electrode. The EMF outputs were plotted against values of pH (Figure 5). As shown in these curves potential readings weren’t stable mostly but shown a distinct stability at 6.5–7.5 pH range and hence 30 mM Tris buffer (pH 7) with 0.01 M Na_2_SO_4_ to adjust the ionic strength was chosen for subsequent potentiometric measurements.

**Figure 6:**
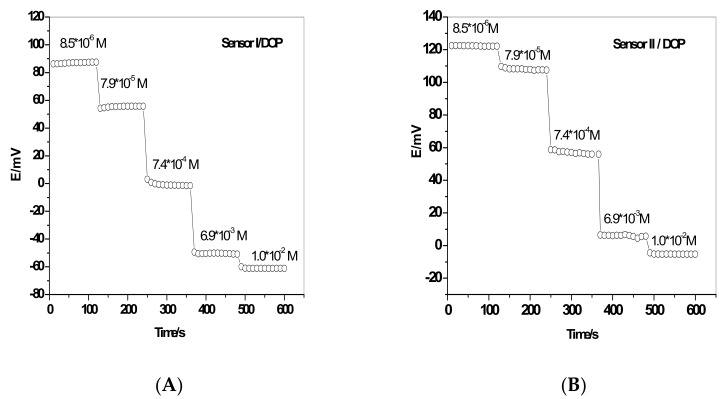
Responses of the proposed sensors using DOP as a plasticizer for (**A**) sensor I and (**B**) sensor II.

The dynamic response time of ion association complexes-based sensors was examined by measuring time to reach a steady and stable potential using 10-fold different concentrations of standard 2,6-DCPIP solutions (Figure 6). The response time of the proposed sensors was <20 s to reach ~95% of equilibrium response for 1.0 × 10^−3^–1.0 × 10^−5^ M 2,6-DCPIP solution.

The selectivity coefficients of the proposed sensors were examined and summarized in Table 3. Applying “the fixed interfering method” [47], using Equation (1):

*K ^pot^_A,B_* = *(a_A_)/(a_B_)^ZA/ZB^* (1)

where *a_A_* is the activity of 2,6-DCPIP when the *EMF* reaches saturation value determined by the value of *a_B_* which represents the constant activity of interfering ions.

As shown in Table 3, Sensor I based on *o*-NPOE as a plasticizer revealed better selectivity than the sensor based on DOP over Cl^−^, Br^−^, NO_2_^−^,NO_3_^−^, acetate, phenol, I^−^, SCN^−^ and pyrogallol. On the other hand, DOP-based sensor revealed better selectivity than *o*-NPOE-based sensor over citrate, PO_4_^3−^, 2,4-DCP, ferulic acid, caffeic acid, gallic acid and catechol. For sensors II based on DOP plasticizer enhanced selectivity was revealed over almost of the selected interfering species except NO_2_^−^, NO_3_^−^, citrate and SCN^−^.

From the results presented above, we can conclude that sensors based on DOP are recommendable over *o*-NPOE ones for almost DOP are recommendable over *o*-NPOE ones for almost all of the studied interfering ions. On the other hand, we draw readers’ attention to: (i) strong interference from acetate and (ii) fact that chloride, bromide and nitrite (in some sensors) interfere more than nitrate. In other words, the selectivity series deviate from that of Hofmeister’. Thus, the invented ion-association complexes act as charged ionophores, and this is interesting.

### 3.3. Potentiometric Determination of Phenolic Antioxidant Compounds

In accordance with the following reaction equation, 1 mole of DCPIP oxidizes two phenolic OH groups: 
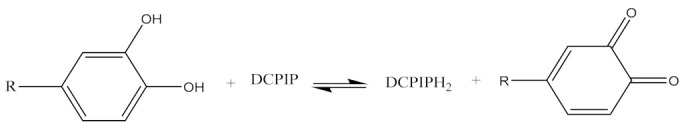


The DCPIP membrane sensor based on ([Cu(Neocup)_2_][DCPIP]_2_ was also used for monitoring some phenolic antioxidants (e.g., Cat, Caf, Pyr, Gal or Fer), singly or in binary and ternary mixtures, with a standard DCPIP solution. According to the oxidation potentials of each phenolic compound, all are oxidized by DCPIP at pH = 6.5. It was found that either Cat or Caf consume 1 mol of DCPIP in which the two phenolic OH groups are oxidized by DCPIP. For ferulic acid (Fer), 1 mol of DCPIP consumes 2 mol of Fer. This is attributed to the presence of only one phenolic OH group. On the other hand, 2 mol of Pyr or Gal consume 3 mol of DCPIP due to the presence of three phenolic OH groups. Table 4 presents results obtained for determination of some mixtures of phenolic antioxidants. The mean average recoveries calculated for the pooled data for these singly or in binary and ternary mixtures are also shown in Table 4.

### 3.4. Analytical Applications

The proposed sensors were applied to the assessment of total antioxidant capacities (TACs) in different juice samples collected from local markets. These samples may contain either natural juices or have flavors and aromas extracted from natural fruits. These natural aromas and flavors are responsible for the antioxidant properties. The obtained results for the collected samples showed different antioxidant capacities. All are expressed in terms of ascorbic acid (AA) concentration as shown in Table 5.

The proposed method is based on using two concentrations (1.0 × 10^−3^ and 1.0 × 10^−4^ M) of 2,6-DCPIP redox dye in titrating the total content of ascorbic acid and phenolic antioxidants in the desired samples. Titration curves were constructed by plotting potential readings against volume of the dye. A pink color which persists for 30 s after the addition of one drop of the 2,6-DCPIP dye is considered the end point of the titration. From the results shown in Table 5, fresh lemon and orange juices are the ones producing the higher antioxidant capacities and canned juice with low concentration content (~10%) the lowest values. The presence of TAC in natural juices or canned juices may result from naturally occurring antioxidants or others formed during its processing/storage. For comparison, the samples were analyzed by the standard DPPH method. For According to this, there is no remarkable difference between the performance of the standard and the proposed methods. The proposed sensors show a good applicability in the assessment of TACs content in real samples.

## 4. Conclusions

A simple low-cost potentiometric membrane sensor for static and hydrodynamic monitoring of 2,6-DCPIP is presented. The potentiometric membranes are based on the incorporation of the ion association complexes of 2,6-DCPIP with either neocuproin/Cu^2+^ and methylene blue. The sensors showed fast response, good selectivity, and compatibility with automated systems. Optimization of the proposed method in addition to its validation for the assay of 2,6-DCPIP enables accurate, precise, and fast assay of 2,6-DCPIP levels as low as 7.72 µg/mL. The great advantage of these membranes is their applicability in estimation of total antioxidant capacity (TAC) in fresh and canned juice samples without any further pre-treatment. pH adjustment may be sometimes necessary. The estimated TAC values determined by the proposed method were compared with the standard DPPH method ones. According to the values obtained, there is no remarkable difference between the performances of the two methods. This is due to the fact that the potentiometric response of the proposed sensors is influenced strongly by the oxidation of the total antioxidants present in the sample by DCPIP reducing dye. Overall the presented method is precise, cheap, characterized by small volumes of reagents used, simple instrumentation, and ease of manipulation.

## Figures and Tables

**Table 1:** Effect of plasticizer on the potentiometric characteristics of 2,6-DCPIP membrane-based sensors.

Parameter	Sensor I/*o*-NPOE	Sensor I/DOP	Sensor II/*o*-NPOE	Senso II/DOP
Slope (mV/decade)	−82.25 ± 1	−56.25 ± 1.7	−59.5 ± 1.4	−51.64 ± 2
Correlation coefficient (r)	−0.996	−0.998	−0.999	−0.998
Linear range (M)	9.9 × 10^−5^−1.0 × 10^−2^	5.13 × 10^−5^−1.0 × 10^−2^	6.5 × 10^−5^−1.0 × 10^−2^	5.15 × 10^−5^−1.0 × 10^−2^
Detection limit (M)	7.9 × 10^−5^	2.37 × 10^−5^	4.5 × 10^−5^	3.45 × 10^−5^
Working range (pH)	7	7	7	7
Response (s)	<20	<20	<20	<20
Life span (week)	12	6	12	6
Standard deviation, σ_v_ (mV)	0.58	1.15	1	1.5
Accuracy (%)	99.3	97.6	96	96.8
Precision (%)	0.6	0.8	1.7	1.1

**Table 2:** Performance characteristics of 2,6-DCPIP membrane sensors under hydrodynamic mode of operation in 30 mM Tris buffer (pH 7.0).

Parameter	Sensor I/DOP	Sensor II/DOP
Slope, mV/decade *	−42.3 ± 1.1	−33.3 ± 0.9
Correlation coefficient, r	−0.974	−0.984
Detection limit, M	8.06 × 10^−5^	4.23 × 10^−5^
Linear range, M	9.8 × 10^−5^−1.0 × 10^−2^	1.0 × 10^−4^−1.0 × 10^−2^
Life span, week	12	12
Optimum flow rate, mL/min	3.5	3.5
Sample frequency, sample/h	51	42

* Average of five measurements.

**Table 3:** Selectivity coefficients (log *K_DCPIP, J_*) ± SD obtained for the proposed sensors.

Interfering ion, *J*	(log *K_DCPIP, J_)* *±* *SD*			
	Sensor I		Sensor II	
	*o*-NPOE	DOP	*o*-NPOE	DOP
Cl^−^	−3.05 ± 0.1	−2.15 ± 0.2	−2.7 ± 0.2	−2.9 ± 0.3
Br^−^	−3.0 ± 0.4	−2.1 ± 0.5	−2.5 ± 0.3	−3.1 ± 0.1
NO_2_^−^	−2.9 ± 0.1	−2.3 ± 0.4	−2.2 ± 0.4	−1.1 ± 0.2
NO_3_^−^	−2.8 ± 0.3	−2.5 ± 0.3	−2.4 ± 0.4	−1.5 ± 0.6
Citrate	−3.2 ± 0.4	−3.4 ± 0.1	−3.6 ± 0.3	−3.5 ± 0.2
Acetate	−1.01 ± 0.2	−0.9 ± 0.07	−0.7 ± 0.03	−0.68 ± 0.02
PO_4_^3−^	−3.4 ± 0.4	−3.6 ± 0.4	−3.3 ± 0.3	−3.7 ± 0.4
Phenol	−2.6 ± 0.1	−2.37 ± 0.3	−1.1 ± 0.2	−1.93 ± 0.1
Picrate	−0.8 ± 0.04	−0.75 ± 0.03	−0.75 ± 0.03	−0.63 ± 0.03
2,4-DCP	−1.3 ± 0.2	−2.7 ± 0.2	−1.2 ± 0.1	−1.8 ± 0.4
I^−^	−2.65 ± 0.1	−1.1 ± 0.1	−1.01 ± 0.2	−1.3 ± 0.2
SCN^−^	−2.5 ± 0.2	−1.5 ± 0.2	−2.55 ± 0.2	−1.02 ± 0.3
Pyrogallol	−2.2 ± 0.3	−1.8 ± 0.2	−1.5 ± 0.1	−2.1 ± 0.1
Ferulic acid	−2.3 ± 0.2	−2.8 ± 0.1	−1.6 ± 0.3	−2.5 ± 0.2
Caffeic acid	−2.35 ± 0.1	−2.8 ± 0.2	−1.7 ± 0.2	−2.4 ± 0.4
Gallic acid	−2.4 ± 0.2	−2.65 ± 0.3	−1.65 ± 0.2	−2.2 ± 0.2
Catechol	−2.01 ± 0.2	−2.55 ± 0.2	−1.77 ± 0.4	−2.47 ± 0.2

**Table 4:** Estimation of phenolic antioxidants quantities in mixtures using DCPIP-based membrane sensor.

Mixture	Added (µg/mL)	Sensor I/*o*-NPOE			Sensor I/DOP		
		Found (µg/mL)	Recovery (%)	S.D. (%)	Found (µg/mL)	Recovery (%)	S.D. (%)
Ferulic acid (Fer)	100	93.3 ± 7.2	93.3	1.2	94.1 ± 3.1	97.1	1.1
Catechol (Cat)	100	97.5 ± 3.5	97.5	0.9	96.7 ± 0.9	96.7	0.9
Caffeic acid (CA)	100	104.0 ± 1.5	104.0	0.7	99.1 ± 0.3	99.1	0.4
Gallic acid (GA)	100	94.0 ± 2.5	94.0	0.9	95.1±1.1	95.1	0.6
Pyrogallol (Pyr)	100	91.2 ± 6.5	91.2	0.3	93.7 ± 2.1	93.7	0.5
Ascorbic acid (AA)	100	102.3 ± 9.5	102.3	0.6	98.2 ± 2.4	98.2	1.4
Cat + Fer	200	214.2 ± 4.5	107.2	0.5	208.1 ± 3.1	104.0	1.3
Fer + Pyr	200	211.5 ± 6.5	105.7	0.6	207 ± 2.4	103.5	0.6
Caf + Gal	200	184 ± 3.5	92.0	1.2	194.2 ± 3.1	97.1	1.3
Cat + Fer + Pyr	300	294.3 ± 1.5	98.1	1.1	291.1 ± 7.1	97.0	0.7

**Table 5:** Potentiometric assessment of TAC with 2,6-DCPIP-based sensors in commercial, fresh juices and pharmaceutical drugs.

Sample		TAC, µg/mL * (AA)		
		DPPH Standard method [45]	Sensor I/DOP	Sensor II/DOP
**1-Vitacid ^a^** (Chemical Industries Development (CID), Egypt)		1005.1 ± 3.8	980.7 ± 3.2	1010.1 ± 7.7
**2-C-Vit ^b^** (Universal Pharmaceutical Industries (Unipharma), Egypt)		1010.2 ± 11.2	1050.3 ± 2.7	1062.6 ± 5.5
**3-Canned orange juices**	**1-Juhayna Pure** (El Dawleya Co. for Modern Food Industries, Egypt) (100%)	513.6 ± 10.1	492.5 ± 6.6	483.8 ± 3.7
	**2- Juhayna** (El Dawleya Co. for Modern Food Industries, Egypt) (˂25%)	281.8 ± 5.4	255.2 ± 3.2	247.3 ± 7.1
	**3-Domty** (Arabian Food Industries, Egypt) (˂25%)	424.5 ± 8.3	410.3 ± 5.9	371.7 ± 6.3
	**4-Faragello Gold** (The Egyptian Food Company, Egypt) (100%)	157.7 ± 7.2	142.6 ± 7.1	139.4 ± 5.2
	**5- Faragello** (The Egyptian Food Company, Egypt) (10%)	37.1 ± 1.8	28.2 ± 2.2	30.4 ± 2.1
	**6- Easymoozoo** (The Egyptian Food Company, Egypt) (10%)	48.9 ± 5.6	36.4 ± 1.4	41.1 ± 1.9
	**7- Prego** (Brego for Food Industries, Egypt) (25%)	104.9 ± 6.3	97.1 ± 1.2	91.2 ± 3.7
**8- Lipton iced tea** (Pepsi Bugshan Investment, a SAE Unilever trademark) orange juice?		95.9 ± 8.1	83.5 ± 2.4	81.4 ± 5.6
**4-Fresh Lemon juice**		378.6 ± 9.5	350.2 ± 4.2	361.3 ± 6.2
**5- Fresh Orange juice**		77.6 ± 2.4	66.1 ± 1.1	61.3 ± 3.7

^a^ labeled 1 g/tablet, ^b^ labeled 100 mg/1 mL, * Average of five measurements.
